# Workplace Rehabilitation and Supportive Conditions at Work: A Prospective Study

**DOI:** 10.1007/s10926-012-9391-z

**Published:** 2012-10-13

**Authors:** Linda Ahlstrom, Mats Hagberg, Lotta Dellve

**Affiliations:** 1 Department of Public Health and Community Medicine, The Sahlgrenska Academy, University of Gothenburg, Medicinaregatan 16, P.O. Box 414, 405 30 Gothenburg, Sweden; 2 Department of Ergonomics, KTH - Royal Institute of Technology, 141 52 Stockholm, Sweden; 3 Health Sciences, University of Borås, 501 90 Borås, Sweden

**Keywords:** Sickness absence, Back to work, Female, Rehabilitation activity, Self-reported, Working degree, Occupational health

## Abstract

*Purpose* To investigate the impact of rehabilitation measures on work ability and return to work (RTW), specifically the association between workplace rehabilitation/supportive conditions at work and work ability and RTW over time, among women on long-term sick leave. *Methods* Questionnaire data were collected (baseline, 6 and 12 months) from a cohort of women (*n* = 324). Linear mixed models were used for longitudinal analysis of the repeated measurements of work ability index (WAI), work ability score and working degree. These analyses were performed with different models; the explanatory variables for each model were workplace rehabilitation, supportive conditions at work and time. *Results* The individuals provided with workplace rehabilitation and supportive conditions (e.g. influence at work, possibilities for development, degree of freedom at work, meaning of work, quality of leadership, social support, sense of community and work satisfaction) had significantly increased WAI and work ability score over time. These individuals scored higher work ability compared to those individuals having workplace rehabilitation without supportive conditions, or neither. Additionally, among the individuals provided with workplace rehabilitation and supportive conditions, working degree increased significantly more over time compared to those individuals with no workplace rehabilitation and no supportive conditions. *Conclusion* The results highlight the importance of integrating workplace rehabilitation with supportive conditions at work in order to increase work ability and improve the RTW process for women on long-term sick leave.

## Introduction

Management of return to work (RTW) is important and is currently a priority on the social and political agenda in Sweden. There is a great interest in providing rehabilitation measures and supportive conditions at work for the individual that promote a sustainable and healthy working life. This study contributes to this by focusing on the importance of the interaction between rehabilitation measures and supportive conditions for increased work ability and RTW among women on long-term sick leave.

The process of RTW following long-term sick leave is particularly challenging. Successful methods of promoting RTW include rehabilitation measures within a multidisciplinary team [1, 2], structured programs [3], and clear goals and milestones [4, 5]. The involvement and participation of the workplace is crucial in order for a positive rehabilitation process, increased work ability and RTW for the individual [6–8]. Work accommodation needs to occur and there should be interaction between stakeholders [9]. Studies have revealed that often workplace rehabilitation and training are not used to a sufficient degree and rehabilitation measures are put into place too late in the rehabilitation process [10]. A review by Shaw et al. [11] concludes that RTW depends more on work accommodation (ergonomics), communication, and conflict resolution rather than medical training and treatment.

Health supportive conditions that are related to successful RTW are: high life satisfaction [12]; a high sense of coherence[12, 13]; balance in life [14]; meaningful work tasks [15]; meaningful activities outside of work; as well as social support both at work and outside work [1, 14, 16, 17]. Studies find that interventions which combine comprehensive health promotion, condition-focused rehabilitation, and secondary prevention in risk groups among those with poor health lead to the best outcomes in terms of clinical conditions and cost effectiveness [18]. However, research and practice regarding health supportive conditions and secondary prevention are seldom integrated. Additionally there is a need to study the differences in individuals’ reasons for being on sick leave in order to tailor an appropriate program [18]. For example, female workers have a higher prevalence of sick leave, especially long-term sick leave and permanent work disability, than men due to musculoskeletal and mental health disorders [19–23] and women will require a different program than male workers. In Sweden, women and men work in different sectors of the labour market.

There is no consensus on how to measure and evaluate the result of RTW. Earlier studies have used a number of outcome measures such as work ability, working degree, and sick leave status. Work ability is the ability of a worker to perform her job, taking into account the specific work demands, individual health conditions, and mental resources [24]. Ilmarinen et al. [25] used linear regression models to show that work ability could be explained by the interaction of health and functional capacities with factors of working life. Sick leave status is most often measured as either presence or absence from work, progress of RTW from long-term sick leave is often more characterized by “small steps” of increased work ability and working degree [26].

There is a need of more in depth knowledge about the association between rehabilitation measures and supportive conditions at the work place that could increase work ability and RTW. There are various forms of rehabilitation measures and supportive conditions within the rehabilitation process, although it has been difficult to know which is most effective. The purpose of this study was to investigate the impact of rehabilitation measures on work ability and RTW, specifically the association between workplace rehabilitation/supportive conditions at work and work ability and RTW over time, among women on long-term sick leave.

## Method

### Study Design

This study consisted of a cohort of women (*n* = 324) working within human service organizations (HSO) and on long-term sick leave at the start of recruitment. Questionnaire data were collected at baseline, 6 and 12 months. The project was approved by the Ethics Committee of Gothenburg University.

### Sickness Insurance System

In Sweden, the sickness insurance system covers the entire working population. The principle is to provide compensation through sickness benefits when a worker has decreased work ability due to sickness or injury (i.e. medical conditions) and is not able to work. A worker needs to lose at least 25 % of their work ability in order to be eligible for benefits from sickness insurance and it is possible to receive sickness benefits covering 25, 50, 75 or 100 % of the working time. During the study period the sickness length was almost unlimited, but now it is limited to 1 year (with the exception of certain diagnoses, such as cancer). Swedish employers have broadly legislated responsibility to rehabilitate and adapt work to the worker in order to allow RTW.

### Study Sample

Data collection began in August 2005 among a cohort of women working within a HSO, on long-term sick leave and employed by one of Sweden’s three largest metropolitan cities [26]. Council employees on long-term sick leave were invited to take part in the study. The inclusion criteria were: female, aged 35–65 years, and currently on long-term sick leave (>60 days) to a degree of at least 50 %. The council employer at the time of the study had 633 individuals fulfilling these criteria and provided the postal addresses. Individuals that replied and chose to participate received the baseline questionnaire. There were 324 individuals that responded and they were mailed follow-up questionnaires at 6 and 12 months; non-respondents were sent one reminder letter. The response rate was 72 % (*n* = 233) in the second wave and 60 % (*n* = 194) in the third wave.

Most study participants (43 %) were between 45 and 55 years of age; around a third (27 %) were older and another third (29 %) younger (Table 1). At baseline, most participants (71 %) worked 25 % or less and more than half of the total group (63 %) had been on sick leave for more than a year. At the 6-month follow-up, 80 % were still on sick leave for more than 25 % of the time while 16 % were back to work full time. The corresponding figures for the 12-month follow-up were 65 % on >25 % sick leave and 24 % back full time. Most participants had musculoskeletal (48 %) and/or mental health (40 %) diagnoses. More than a third (39 %) worked within schools or preschool care, almost a third (30 %) worked in elderly/home care, while others worked in the areas of caring for disabled persons/personal assistance (16 %), administrative work (6 %), food/cleaning (4 %), and unspecified other work (5 %).

**Table 1:** Descriptive data of rehabilitation measures among women on long-term sick leave in relation to age group, working degree, length of sick leave, different diagnosis and type of work at baseline

Baseline	All	Workplace rehabilitation	Offsite occupational rehabilitation	Physiotherapy	Medical treatment	Self-directed physical exercise	Rehabilitation courses/programs	Socio/psychotherapy	Complementary medicine
	*n*	*n* (%)	*n* (%)	*n* (%)	*n* (%)	*n* (%)	*n* (%)	*n* (%)	*n* (%)
All	324	79 (24)	51 (16)	221 (68)	271 (83)	262 (80)	90 (28)	164 (50)	149 (46)
Age group (years)^a^									
35–44	88	17 (19)	13 (15)	63 (72)	74 (84)	69 (78)	26 (30)	45 (51)	38 (43)
45–54	139	40 (29)	21 (15)	91 (66)	115 (83)	114 (82)	40 (29)	80 (58)	71 (51)
≥55	93	21 (23)	16 (17)	65 (69)	80 (86)	77 (83)	24 (26)	38 (41)	39 (42)
Working degree (%)^a^									
100–86	25	7 (28)	1 (4)	14 (56)	17 (68)	19 (76)	2 (8)	11 (44)	8 (32)
85–51	30	7 (23)	3 (10)	21 (70)	24 (80)	21 (70)	7 (23)	12 (40)	13 (43)
50–26	23	6 (26)	5 (22)	16 (70)	19 (83)	20 (87)	5 (22)	11 (48)	11 (48)
<26	230	55 (24)	40 (17)	158 (69)	197 (86)	191 (83)	73 (32)	121 (53)	110 (48)
Length of sick leave (year)^a^									
≤1	106	21 (20)	4 (4)	70 (66)	80 (76)	72 (68)	12 (11)	40 (38)	34 (32)
>1	204	56 (28)	45 (22)	142 (70)	179 (88)	178 (87)	77 (38)	114 (89)	108 (53)
Diagnosis/disorder^b^									
Musculoskeletal	154	36 (23)	25 (16)	124 (80)	136 (88)	130 (84)	44 (28)	69 (45)	84 (55)
Mental health	131	32 (28)	23 (20)	79 (60)	112 (86)	107 (82)	37 (28)	87 (77)	59 (52)
Cardiac	25	8 (32)	5 (20)	17 (69)	22 (88)	22 (88)	6 (24)	12 (48)	10 (40)
Pulmonary	30	13 (43)	5 (20)	18 (60)	24 (80)	23 (77)	8 (27)	12 (40)	16 (53)
Musculoskeletal and mental health	106	24 (23)	18 (17)	76 (72)	94 (89)	88 (83)	35 (33)	70 (66)	59 (56)
Type of work^a^									
Elderly and homecare	97	24 (25)	15 (16)	72 (74)	81 (84)	80 (83)	28 (29)	41 (42)	42 (43)
Preschool care	63	22 (35)	9 (14)	45 (71)	57 (91)	51 (81)	19 (30)	31 (49)	32 (51)
Care of disabled	26	7 (27)	6 (23)	17 (66)	22 (85)	19 (73)	7 (27)	14 (54)	10 (39)
School	63	10 (16)	10 (16)	41 (65)	51 (81)	52 (83)	20 (32)	32 (51)	32 (51)
Administration	50	12 (24)	9 (18)	27 (54)	42 (84)	38 (76)	15 (30)	31 (62)	22 (44)
Unspecified other	40	10 (25)	3 (8)	25 (63)	31 (78)	33 (83)	6 (15)	22 (55)	17 (43)

% numbers of rehabilitation measures out of total of all individuals in that group

^a^Numbers do not add up to (all) *n* = 324 due to missing data

^b^Numbers do not add up to (all) *n* = 324 due to that all types of diagnosis/disorders are not presented, and individuals could have more than one diagnose/disorder

All studied characteristics of the group (Tables 1, 2) were analysed at baseline to examine possible differences between drop-outs at follow-up and those participants fulfilling the study, and no significant differences (*p* < 0.05) were found. For the individuals (*n* = 309) that chose not to participate at all we succeeded to retrieve data from the employer for 186 individuals (60 %). Of those individuals 59 % were on full-time sick leave and the rest 41 % on part-time sick leave, in the participating group 72 % of the individuals were on full-time sick leave. The age of the individuals among the non-participants is consistent with the individuals that chose to participate, as well the proportion of individuals being on sick leave <1 year and more than 1 year were equal.

**Table 2:** Work ability index (WAI), work ability score and working degree (%) in relation to rehabilitation measure and number of rehabilitation measures over time (baseline, 6 and 12 months) for women on long-term sick leave

	Work ability index (WAI)				Work ability score				Working degree (%)			
	*n*	Baseline	6 month	12 month	*n*	Baseline	6 month	12 month	*n*	Baseline	6 month	12 month
		*m*	*m*	*m*		*m*	*m*	*m*		*m*	*m*	*m*
All	290	23.7	26.7^a^	27.7^b^	315	3.9	4.8^a^	5.1	308	19.6	45.2^a^	52.2^b^
Rehabilitation measure												
Workplace rehabilitation	73	23.3	27.0^a^	29.5^b^	78	4.0	5.1^a^	5.7^b^	79	20.3	46.3^a^	54.2^b^
Offsite occupational rehabilitation	48	21.7	23.7^a^	25.5^b^	50	3.6	4.3^a^	4.3^b^	49	11.7	32.6^a^	44.5^b^
Physiotherapy	198	22.5	25.8^a^	26.4^b^	215	3.7	4.6^a^	4.8^b^	221	18.8	42.1^a^	48.9^b^
Medical treatment	242	23.2	26.3^a^	27.5^b^	263	3.8	4.8^a^	5.0^b^	271	17.9	43.4^a^	52.2^b^
Self-directed physical exercise	236	23.2	26.2^a^	27.4^b^	255	3.8	4.7^a^	5.0^b^	262	18.2	43.8^a^	51.6^b^
Rehabilitation courses/programs	80	22.2	25.2^a^	26.0^b^	88	3.8	4.6^a^	4.8^b^	90	12.2	39.1^a^	48.7^b^
Socio/psychotherapy	148	22.6	25.6^a^	26.5^b^	158	3.6	4.6^a^	4.7^b^	164	16.7	42.5^a^	51.2^b^
Complementary medicine	136	22.0	24.9^a^	27.3^b^	144	3.6	4.5^a^	5.0^b^	142	16.6	42.1^a^	54.0^b^
Number of rehabilitation measures												
1–2	49	25.9	29.7^a^	33.0^b^	52	4.3	5.3^a^	6.4^b^	51	19.3	50.2^a^	53.13
3–5	175	23.7	26.9^a^	27.7^b^	194	3.9	4.8^a^	5.0^b^	191	20.9	46.3^a^	52.90^b^
6–8	59	20.4	23.9^a^	25.8^b^	60	3.3	4.5^a^	4.7^b^	58	9.1	35.8^a^	49.48^b^

^a^Increased (baseline-6 month)/Wilcoxon signed rank *p* < 0.05

^b^Increased (baseline-12 month)/Wilcoxon signed rank *p* < 0.05

### Questionnaire Data

Rehabilitation measures were assessed by self-report of whether they took part (yes/no) in the activity [27]. Questions were classified into a number of categories: medical treatment (physician/hospital care), physiotherapy, self-directed physical exercise, courses/programs (back/neck school or psychologically/socially-focused rehabilitation, comprehensive rehabilitation program for 4 h per day over a period of at least 4 days), socially/psychologically-focused rehabilitation (psychologist/welfare officer), complementary medicine (acupuncture, chiropractic, and/or naprapathy), workplace rehabilitation (at the workplace, mainly including work training, assessment of work capacity and individually supportive actions, physical and psychosocial changes in the work environment, organization, work tasks, working hours and distribution of work), and offsite occupational rehabilitation (external to the workplace).

Work ability index (WAI) is a summary measure of seven items (10 questions): current work ability compared with lifetime best, work ability in relation to the demands of the job, number of current diseases diagnosed by a physician, estimated work impairment due to diseases, sick leave during the past year (12 months), own prognosis of work ability 2 years from now, and mental resources [28–32]. Although WAI has been validated [28, 33], it includes items of both changeable and non-changeable status. Therefore, as an additional outcome measure, we used the recently validated work ability score [26], i.e. current work ability compared with lifetime best (score = 0–10).

Working degree ranged from 0 to 100 %, based on the response to one item in the questionnaire: “What is your current work status?” The possible responses were: (1) on full-time/part-time sick leave; (2) on full-time/part-time temporary disability pension; and (3) working full-time/part-time. The response specified the percentage of each status and the starting date of the current status. Where possible (in two-thirds of cases), these self-reported data were compared with employers’ register-based data and medical records, in order to check the validity of the self-reported data. We found no important discrepancies and this helped to add missing data. In this study the definition of RTW is the outcome of working degree.

The sense of feeling welcome back at work was measured with one item: “Do you feel that you were welcome back to work?” with response alternatives of “yes, fully”, “yes, partly” and “no”. A positive response to this item was defined as an answer of “yes, fully” [34].

The Copenhagen Psychosocial Questionnaire (COPSOQ), which is a valid and reliable tool [35], was used to measure the psychosocial environment of the workplace. Items used were quantitative demands (3 items), emotional demands (3 items), influence at work (3 items), possibilities for development (2 items), degree of freedom at work (1 item), meaning of work (2 items), quality of leadership (4 items), social support (2 items), sense of community (2 items), and work satisfaction (4 items). Each item on this scale has 4 or 5 graded responses which are then recalculated to 0–100 points. When dichotomising, we split at the neutral value (50 points) except for the variable “meaning of work”, where we used a median split (>75) due to skewed distribution of the data. The variables sense of feeling welcome back at work and COPSOC variables will be referred to as supportive conditions at work.

### Statistical Analysis

Descriptive analysis of each variable was made. Then, a cross-sectional analysis with prevalence ratios (PR) was used to examine possible relationships between the explanatory factors and the outcomes. PRs were calculated according to the research questions and correlations between the risk factors were tested. Finally, linear mixed models were used for longitudinal analysis of the repeated measurements of WAI, work ability score and working degree. These analyses were performed with different models, the explanatory variables for each model were workplace rehabilitation (yes/no), one of the 11 different supportive conditions at work (yes/no) and time (baseline, 6 and 12 month). WAI and work ability score were assumed to be continuous and not ordinal variables. Data for assessment were assumed to be normally distributed. All Least squares means analyses were statistically significant at *p* ≤ 0.001. Data were analysed using version 9 of the JMP^®^ software package and SAS version 9.3 for the linear mixed models (SAS Institute Inc., Cary, NC, USA).

## Results

The most frequently used rehabilitation measures among women on long-term sick leave were medical treatment, self-directed physical exercise and physiotherapy (Table 2). About half of the study population had participated in socially/psychologically-focused rehabilitation and/or complementary medicine. A third of them had participated in rehabilitation courses/programs. A quarter of the individuals had participated in rehabilitation at the workplace while only a few had participated in offsite occupational rehabilitation. The distribution of rehabilitation measures was relatively equal among the different age groups except that rehabilitation at the workplace and socially/psychologically-focused rehabilitation were more common among the middle age group (45–54 years).

The individuals on sick leave for longer than a year reported more participation in rehabilitation courses/programs (PR [95 % CI]: 3.33 [1.90; 5.84]) and in socially/psychologically-focused rehabilitation (PR [95 % CI]: 1.48 [1.13; 1.95]) compared to their counterparts on sick leave for less than a year.

### Rehabilitation, Work Ability and RTW

All rehabilitation measures were positively related to increased WAI, increased work ability score and increased working degree at both the 6- and 12-month follow-up (Table 3) among these women on long-term sick leave. The individuals who underwent workplace rehabilitation had the greatest increase in work ability and working degree at both follow-ups. Participation in workplace rehabilitation was positively associated with increased work ability (PR [95 % CI]: 1.78 [1.38; 2.29]) and RTW (PR [95 % CI]: 1.40 [1.09; 1.80]). Additionally, being pleased with the employer’s efforts to help them RTW was positively associated with increased work ability (PR [95 % CI]: 1.91 [1.14; 3.19]).

**Table 3:** Repeated measures ANOVA of work ability index (WAI) among women on long-term sick leave

Model	Work ability index (WAI)			Difference group 1–2	Difference group 1–4
	Least square mean (LSM)			Overtime	Overtime
				Estimate (SE)	Estimate (SE)
Workplace rehabilitation yes/no	Standard error (SE)			*p* value	*p* value
Supportive condition at work yes/no	Baseline	6 month	12 month		
	LSM (SE)	LSM (SE)	LSM (SE)		
Sense of feeling welcome back (SWB)					
1. Workplace rehab + SWB	25.2 (1.3)	29.2 (1.5)	30.0 (1.6)	8.5 (1.9)	9.8 (2.2)
2. Workplace rehab + not SWB	21.5 (1.4)	24.7 (1.7)	26.6 (2.0)	<0.001	0.001
3. No workplace rehab + SWB	25.9 (0.7)	28.5 (0.9)	28.2 (1.1)		
4. No workplace rehab + not SWB	20.2 (0.9)	23.6 (1.2)	24.0 (0.9)		
Quantitative demands (QD)					
1. Workplace rehab + QD	22.5 (1.2)	26.0 (1.4)	26.9 (1.6)	1.7 (2.2)	0.6 (1.8)
2. Workplace rehab + not QD	25.2 (1.6)	29.2 (1.9)	31.3 (2.0)	0.433	0.745
3. No workplace rehab + QD	22.6 (0.7)	25.7 (0.9)	25.8 (1.0)		
4. No workplace rehab + QD	26.3 (1.0)	28.7 (1.3)	28.4 (1.4)		
Emotional demands (ED)					
1. Workplace rehab + ED	22.1 (1.2)	26.7 (1.4)	28.0 (1.5)	1.2 (2.3)	3.2 (1.8)
2. Workplace rehab + not ED	26.8 (1.8)	28.2 (2.1)	29.4 (2.3)	0.597	0.074
3. No workplace rehab + ED	23.5 (0.7)	26.5 (0.9)	26.3 (1.0)		
4. No workplace rehab + not ED	24.8 (1.0)	27.3 (1.4)	27.5 (1.5)		
Influence at work (IW)					
1. Workplace rehab + IW	25.3 (1.3)	29.4 (1.6)	32.0 (1.6)	10.4 (2.1)	10.0 (1.8)
2. Workplace rehab + not IW	21.6 (1.4)	24.8 (1.6)	24.1 (1.8)	<0.001	<0.001
3. No workplace rehab + IW	26.0 (0.8)	29.6 (1.0)	29.5 (1.2)		
4. No workplace rehab + not IW	22.0 (0.7)	24.1 (1.0)	24.0 (1.1)		
Possibilities for development (PD)					
1. Workplace rehab + PD	23.6 (1.0)	27.8 (1.2)	29.1 (1.3)	6.2 (3.1)	10.5 (1.8)
2. Workplace rehab + not PD	22.9 (2.9)	20.9 (3.5)	21.8 (4.6)	0.050	<0.001
3. No workplace rehab + PD	24.9 (0.6)	27.8 (0.8)	27.5 (0.9)		
4. No workplace rehab + not PD	18.6 (1.3)	20.3 (1.9)	22.1 (2.3)		
Degree of freedom at work (DF)					
1. Workplace rehab + DF	24.3 (1.2)	28.3 (1.4)	30.2 (1.5)	7.8 (2.2)	8.4 (1.7)
2. Workplace rehab + not DF	22.3 (1.5)	25.5 (1.7)	26.1 (1.9)	0.001	<0.001
3. No workplace rehab + DF	26.4 (0.9)	30.4 (1.0)	30.9 (1.1)		
4. No workplace rehab + not DF	21.8 (0.7)	23.5 (0.9)	22.8 (1.1)		
Meaning of work (MW)^a^					
1. Workplace rehab + MW	22.8 (1.3)	28.5 (1.5)	29.7 (1.6)	5.2 (2.2)	7.2 (1.8)
2. Workplace rehab + not MW	24.5 (1.5)	25.3 (1.8)	26.7 (2.0)	0.020	<0.001
3. No workplace rehab + MW	24.7 (0.7)	27.7 (0.9)	27.6 (1.0)		
4. No workplace rehab + not MW	22.5 (0.9)	24.7 (1.2)	24.9 (1.5)		
Quality of leadership (QL)					
1. Workplace rehab + QL	23.0 (1.4)	28.5 (1.6)	29.1 (1.8)	4.9 (2.3)	5.4 (1.9)
2. Workplace rehab + not QL	24.2 (1.4)	26.4 (1.7)	28.4 (1.8)	0.035	0.006
3. No workplace rehab + QL	24.2 (1.0)	27.6 (1.1)	27.0 (1.3)		
4. No workplace rehab + not QL	23.6 (0.7)	26.1 (1.0)	26.4 (1.1)		
Social support (SS)					
1. Workplace rehab + SS	24.0 (1.3)	28.2 (1.5)	29.3 (1.6)	6.5 (2.2)	6.2 (1.8)
2. Workplace rehab + not SS	22.8 (1.5)	25.6 (1.8)	27.3 (2.0)	0.004	0.001
3. No workplace rehab + SS	24.6 (0.8)	27.2 (1.0)	27.1 (1.1)		
4. No workplace rehab + not SS	23.1 (0.8)	26.3 (1.1)	26.2 (1.3)		
Sense of community (SC)					
1. Workplace rehab + SC	24.2 (1.1)	28.1 (1.2)	28.7 (1.4)	8.7 (2.6)	8.9 (1.8)
2. Workplace rehab + not SC	20.0 (2.2)	22.3 (2.7)	27.2 (2.9)	0.001	<0.001
3. No workplace rehab + SC	25.3 (0.6)	27.8 (0.8)	27.9 (0.9)		
4. No workplace rehab + not SC	19.8 (1.1)	23.5 (1.5)	22.7 (1.7)		
Work satisfaction (WS)					
1. Workplace rehab + WS	24.8 (1.0)	28.2 (1.2)	30.4 (1.3)	11.3 (2.4)	12.2 (1.7)
2. Workplace rehab + not WS	19.0 (2.0)	23.8 (2.5)	21.0 (2.7)	<0.001	<0.001
3. No workplace rehab + WS	25.7 (0.6)	29.0 (0.8)	29.1 (0.9)		
4. No workplace rehab + not WS	18.3 (1.0)	19.3 (1.4)	17.8 (1.7)		

In each of the models supportive condition at work (yes/no), workplace rehabilitation (yes/no) and time are explanatory variables

^a^Cut-off ≥75

### Supportive Conditions at Work and Workplace Rehabilitation

At baseline, most participants (65 %) were wholly or partly dissatisfied with their employers’ efforts to help them RTW and one in four (24 %) were not satisfied at all. The individuals provided with workplace rehabilitation and supportive conditions at work such as influence at work, possibilities for development, degree of freedom at work, meaning of work, quality of leadership, social support, sense of community and work satisfaction, increased in WAI significantly more over time and scored higher WAI in comparison to those individuals who had workplace rehabilitation but no such supportive conditions, or neither (Table 3). The group result strongly coincided for the outcome work ability score (Table 4). Additionally, the individuals provided with workplace rehabilitation and supportive conditions increased working degree significantly more over time (*p* < 0.001) compared to those individuals with no workplace rehabilitation and no supportive conditions (Table 5).

**Table 4:** Repeated measures ANOVA of work ability score among women on long-term sick leave

Model	Work ability score			Difference group 1–2	Difference group 1–4
	Least square mean (LSM)			Overtime	Overtime
Workplace rehabilitation yes/no	Standard error (SE)				
Supportive condition at work yes/no	Baseline	6 month	12 month	Estimate (SE)	Estimate (SE)
	LSM (SE)	LSM (SE)	LSM (SE)	*p* value	*p* value
Sense of feeling welcome back (SWB)					
1. Workplace rehab + SWB	4.1 (0.4)	5.8 (0.5)	6.3 (0.5)	2.4 (0.7)	3.5 (0.6)
2. Workplace rehab + not SWB	4.5 (0.5)	4.9 (0.6)	4.1 (0.4)	0.001	<0.001
3. No workplace rehab + SWB	4.5 (0.2)	5.2 (0.3)	5.2 (0.3)		
4. No workplace rehab + not SWB	2.8 (0.3)	3.9 (0.4)	3.9 (0.4)		
Quantitative demands (QD)					
1. Workplace rehab + QD	3.9 (0.4)	5.1 (0.4)	5.6 (0.4)	1.4 (0.7)	1.1 (0.5)
2. Workplace rehab + not QD	4.2 (0.5)	5.5 (0.6)	6.1 (0.6)	0.046	0.036
3. No workplace rehab + QD	3.6 (0.2)	4.5 (0.3)	4.3 (0.3)		
4. No workplace rehab + QD	4.4 (0.3)	5.2 (0.4)	5.7 (0.4)		
Emotional demands (ED)					
1. Workplace rehab + ED	3.7 (0.4)	5.2 (0.4)	5.6 (0.4)	1.0 (0.7)	1.8 (3.2)
2. Workplace rehab + not ED	4.7 (0.6)	5.3 (0.6)	6.0 (0.7)	0.200	0.001
3. No workplace rehab + ED	3.9 (0.2)	4.8 (0.3)	4.5 (0.3)		
4. No workplace rehab + not ED	3.8 (0.3)	4.7 (0.4)	5.3 (0.4)		
Influence at work (IW)					
1. Workplace rehab + IW	4.3 (0.4)	5.4 (0.5)	6.6 (0.5)	2.8 (0.7)	3.2 (0.5)
2. Workplace rehab + not IW	3.7 (0.5)	5.0 (0.5)	4.6 (0.5)	<0.001	<0.001
3. No workplace rehab + IW	4.4 (0.3)	5.3 (0.3)	5.3 (0.3)		
4. No workplace rehab + not IW	3.4 (0.2)	4.2 (0.3)	4.2 (0.3)		
Possibilities for development (PD)					
1. Workplace rehab + PD	4.1 (0.3)	5.3 (0.4)	5.9 (0.4)	2.5 (1.0)	3.2 (0.6)
2. Workplace rehab + not PD	3.4 (0.9)	4.2 (1.1)	4.5 (1.3)	0.012	<0.001
3. No workplace rehab + PD	4.1 (0.2)	5.0 (0.2)	5.0 (0.2)		
4. No workplace rehab + not PD	2.6 (0.4)	3.0 (0.6)	3.3 (0.7)		
Degree of freedom at work (DF)					
1. Workplace rehab + DF	4.0 (0.4)	5.2 (0.4)	5.9 (0.5)	1.9 (0.7)	2.6 (0.5)
2. Workplace rehab + not DF	4.0 (0.5)	5.3 (0.5)	5.5 (0.6)	0.005	<0.001
3. No workplace rehab + DF	4.6 (0.3)	5.6 (0.3)	5.7 (0.3)		
4. No workplace rehab + not DF	3.3 (0.2)	4.0 (0.3)	3.9 (0.3)		
Meaning of work (MW)^a^					
1. Workplace rehab + MW	3.7 (0.4)	5.3 (0.5)	6.3 (0.5)	1.8 (0.7)	2.7 (0.5)
2. Workplace rehab + not MW	4.5 (0.5)	5.0 (0.6)	4.9 (0.6)	0.008	<0.001
3. No workplace rehab + MW	4.0 (0.2)	5.0 (0.3)	4.9 (0.3)		
4. No workplace rehab + not MW	3.6 (0.3)	4.2 (0.4)	4.3 (0.4)		
Quality of leadership (QL)					
1. Workplace rehab + QL	3.9 (0.5	5.4 (0.5)	6.1 (0.5)	1.9 (0.7)	2.3 (0.6)
2. Workplace rehab + not QL	4.2 (0.5)	5.2 (0.5)	5.5 (0.5)	0.009	0.013
3. No workplace rehab + QL	4.0 (0.3)	5.0 (0.3)	4.9 (0.4)		
4. No workplace rehab + not QL	3.8 (0.2)	4.6 (0.3)	4.7 (0.3)		
Social support (SS)					
1. Workplace rehab + SS	3.8 (0.4)	5.3 (0.4)	6.0 (0.5)	1.7 (0.7)	2.2 (0.5)
2. Workplace rehab + not SS	4.3 (0.5)	5.1 (0.6)	5.3 (0.6)	0.016	<0.001
3. No workplace rehab + SS	4.0 (0.3)	5.0 (0.3)	4.8 (0.3)		
4. No workplace rehab + not SS	3.8 (0.3)	4.5 (0.3)	4.7 (0.4)		
Sense of community (SC)					
1. Workplace rehab + SC	4.2 (0.4)	4.5 (0.4)	5.8 (0.4)	2.6 (0.8)	3.0 (0.5)
2. Workplace rehab + not SC	3.2 (0.7)	3.8 (0.8)	5.4 (0.8)	0.002	<0.001
3. No workplace rehab + SC	4.2 (0.2)	5.1 (0.2)	5.1 (0.3)		
4. No workplace rehab + not SC	2.8 (0.4)	3.7 (0.5)	3.6 (0.5)		
Work satisfaction (WS)					
1. Workplace rehab + WS	4.1 (0.4)	5.4 (0.4)	6.2 (0.4)	2.4 (0.8)	3.3 (0.5)
2. Workplace rehab + not WS	3.7 (0.7)	4.4 (0.8)	3.7 (0.8)	0.015	<0.001
3. No workplace rehab + WS	4.2 (0.2)	5.3 (0.2)	5.3 (0.2)		
4. No workplace rehab + not WS	2.9 (0.4)	2.9 (0.4)	2.5 (0.5)		

In each of the models supportive condition at work (yes/no), workplace rehabilitation (yes/no) and time are explanatory variables

^a^Cut-off ≥75

**Table 5:** Repeated measures ANOVA of working degree among women on long-term sick leave

Model	Working degree %			Difference group 1–2	Difference group 1–4
	Least square mean (LSM)			Overtime	Overtime
Workplace rehabilitation yes/no	Standard error (SE)				
Supportive condition at work yes/no	Baseline	6 month	12 month	Estimate (SE)	Estimate (SE)
	LSM (SE)	LSM (SE)	LSM (SE)	*p* value	*p* value
Sense of feeling welcome back (SWB)					
1. Workplace rehab + SWB	21.3 (5.3)	52.5 (5.7)	62.6 (6.3)	41.5 (8.8)	51.2 (7.3)
2. Workplace rehab + not SWB	21.1 (6.1)	38.5 (6.7)	41.7 (7.8)	<0.001	<0.001
3. No workplace rehab + SWB	23.6 (2.9)	53.0 (3.5)	55.3 (4.0)		
4. No workplace rehab + not SWB	11.5 (3.7)	32.6 (4.5)	43.1 (5.2)		
Quantitative demands (QD)					
1. Workplace rehab + QD	24.0 (5.1)	42.9 (5.4)	50.6 (6.0)	34.0 (8.7)	24.6 (7.2)
2. Workplace rehab + not QD	16.7 (6.3)	53.3 (7.5)	61.1 (8.1)	0.001	0.001
3. No workplace rehab + QD	16.3 (2.8)	44.8 (3.3)	48.5 (3.9)		
4. No workplace rehab + QD	26.1 (3.9)	48.4 (3.9)	57.0 (5.4)		
Emotional demands (ED)					
1. Workplace rehab + ED	20.5 (4.8)	48.3 (5.2)	51.7 (5.7)	29.8 (9.1)	36.0 (7.1)
2. Workplace rehab + not ED	21.9 (7.0)	40.9 (8.2)	59.8 (9.1)	0.001	<0.001
3. No workplace rehab + ED	21.4 (2.7)	47.3 (3.3)	51.2 (3.7)		
4. No workplace rehab + not ED	15.7 (4.1)	43.2 (5.1)	52.3 (6.1)		
Influence at work (IW)					
1. Workplace rehab + IW	25.8 (5.6)	55.5 (6.0)	65.3 (6.6)	49.0 (8.6)	49.9 (7.2)
2. Workplace rehab + not IW	16.3 (5.5)	36.0 (6.2)	41.6 (7.0)	<0.001	<0.001
3. No workplace rehab + IW	24.2 (3.4)	53.8 (3.9)	56.7 (4.4)		
4. No workplace rehab + not IW	15.4 (2.9)	38.2 (3.8)	46.1 (4.4)		
Possibilities for development (PD)					
1. Workplace rehab + PD	21.8 (4.2)	47.9 (4.5)	54.1 (5.0)	24.4 (15.0)	42.7 (7.3)
2. Workplace rehab + not PD	15.5 (12.0)	29.7 (14.0)	58.4 (17.0	0.003	<0.001
3. No workplace rehab + PD	21.1 (2.5)	50.1 (2.9)	55.4 (3.3)		
4. No workplace rehab + not PD	11.4 (5.4)	18.9 (7.2)	23.5 (8.9)		
Degree of freedom at work (DF)					
1. Workplace rehab + DF	18.3 (5.1)	46.4 (5.5)	57.6 (6.2)	35.6 (8.8)	43.0 (6.8)
2. Workplace rehab + not DF	25.1 (6.2)	46.0 (6.6)	49.2 (7.5)	0.001	<0.001
3. No workplace rehab + DF	26.3 (3.5)	57.5 (3.9)	61.6 (4.5)		
4. No workplace rehab + not DF	14.7 (2.9)	35.9 (3.7)	42.0 (4.4)		
Meaning of work (MW)^a^					
1. Workplace rehab + MW	25.2 (5.2)	51.5 (5.6)	56.7 (6.2)	41.3 (8.7)	38.6 (7.1)
2. Workplace rehab + not MW	15.4 (6.1)	38.9 (6.9)	51.0 (7.9)	<0.001	<0.001
3. No workplace rehab + MW	20.4 (3.0)	49.0 (3.4)	54.8 (3.9)		
4. No workplace rehab + not MW	18.1 (3.5)	40.0 (4.6)	44.2 (5.4)		
Quality of leadership (QL)					
1. Workplace rehab + QL	15.8 (5.6)	43.9 (6.3)	54.3 (7.1)	27.1 (9.2)	35.6 (7.7)
2. Workplace rehab + not QL	27.1 (5.8)	49.2 (6.3)	55.3 (6.9)	0.004	<0.001
3. No workplace rehab + QL	20.3 (3.6)	48.9 (4.2)	51.9 (5.0)		
4. No workplace rehab + not QL	18.7 (2.9)	43.6 (3.7)	50.7 (4.2)		
Social support (SS)					
1. Workplace rehab + SS	15.8 (5.1)	43.6 (5.6)	57.2 (6.4)	28.0 (9.0)	41.6 (7.2)
2. Workplace rehab + not SS	29.1 (6.3)	49.6 (7.0)	50.6 (7.6)	0.002	<0.001
3. No workplace rehab + SS	23.3 (3.1)	50.1 (3.7)	54.4 (4.2)		
4. No workplace rehab + not SS	15.5 (3.2)	41.2 (4.1)	47.7 (4.9)		
Sense of community (SC)					
1. Workplace rehab + SC	21.5 (4.4)	47.8 (4.8)	55.4 (5.3)	10.7 (11.3)	36.1 (6.5)
2. Workplace rehab + not SC	17.1 (9.1)	37.1 (10.3	48.7 (11.3)	0.001	<0.001
3. No workplace rehab + SC	21.9 (2.6)	49.7 (3.1)	55.1 (3.5)		
4. No workplace rehab + not SC	11.8 (4.4)	32.8 (5.9)	37.0 (7.1)		
Work satisfaction (WS)					
1. Workplace rehab + WS	19.8 (4.5)	48.3 (4.7)	60.1 (5.1)	25.0 (11.4)	47.8 (6.7)
2. Workplace rehab + not WS	25.0 (8.3)	35.1 (10.1)	28.8 (10.6)	0.001	<0.001
3. No workplace rehab + WS	22.0 (2.6)	51.5 (3.1)	58.5 (3.4)		
4. No workplace rehab + not WS	12.3 (4.3)	26.2 (5.6)	24.9 (6.5)		

In each of the models supportive condition at work (yes/no), workplace rehabilitation (yes/no) and time are explanatory variables

^a^Cut-off ≥75

When performing the mixed models for different age groups result were younger age groups scored WAI and work ability score slightly higher than the older age groups, although all different age groups presented the same pattern of improvement of these outcomes over time.

## Discussion

This study demonstrates the importance of supportive conditions at work for increasing work ability and working degree among women on long-term sick leave working within HSOs. All rehabilitation measures and, in particular, workplace rehabilitation were associated with small steps towards increased work ability and improved RTW but there was a significantly higher increase when supportive conditions at work were present.

The results highlight the importance of supplementing workplace rehabilitation with supportive conditions at work. Examples of supportive conditions are influence at work, degree of freedom at work, meaning at work, work satisfaction, possibilities for development, quality of leadership, social support, sense of community and feeling welcomed back to work. Earlier studies have identified that factors predicting RTW includes support, level of demands, adjustment, ergonomics, and collaboration between health providers and workplace [5, 36]. These results are in line with the illness flexibility model [5, 37] which highlights the importance of work adjustment for work ability and RTW as well as health supporting interventions at the workplace to improve clinical and cost outcomes [38] among individuals on sick leave. A recent literature review states that implementation of workplace rehabilitation alone in an intervention is not sufficient to enhance RTW among those on long-term (≥2 weeks) sick leave [39]. Working demands and conditions should be adjusted and integrated with the person’s needs, the nature of the work, and the attitudes of the management [40]. Further considering the positive impact of high quality leadership could directly or indirectly influence the individual positively by strengthening the social climate at the workplace [41].

There is a need to find the cause of why an individual remains on sick leave and to consider all the factors involved both workplace and personal factors and the interaction between them [42]. Gzil et al. [43] and Leplege et al. [44] argue that future rehabilitation processes ought to be more person-centred, more focused on patient satisfaction, and aimed at sharing power and responsibility with the individual, in order to enhance the rehabilitation process [45, 46]. A person-centred approach would focus on the person’s ability instead of their actual performance. Tengland [47] argues that while a person might be able to perform and might have the necessary work ability, they may still lack the opportunity, the willingness, or the motivation to go back to their former state of functioning within their former workplace. Nordenfelt [48] emphasizes the importance of society’s responsibility to help and guide individuals and to give them the opportunity to perform and actualize their capacities. In the rehabilitation process it is important to help the individual acknowledge their disability and assist them to recognize their abilities and consider other options and possibilities to allow them to RTW.

The results of this study are consistent with previous research [5], confirming that it is crucial to incorporate the workplace into the rehabilitation process. Research within the area of work disability prevention [11] suggests that the creation of a RTW coordinator role could help ensure a safe and sustainable RTW. Relevant areas for consideration include ergonomic and workplace assessment, workplace mediation, clinical interviewing, social problem solving, and knowledge of medical conditions [9, 11]; these should be treated within a multidimensional perspective [49].

### Methodological Discussion

One limitation of the study is that the rehabilitation measures were grouped in a fairly rough classification and aspects such as the amount, type, and quality of the rehabilitation were not taken into account. Another is that the RTW data were collected for a specific point in time; we do not know what may have happened in between. This is an important point given that individuals may change their type of sick leave frequently. Being on long-term sick leave is itself a predictor for not returning to work [50] so within this study group we can usually only see only small steps of improvement.

There could be an issue when interpreting data due to loss to follow-up, although method chosen when analyzing the data; linear mixed models for longitudinal analyses of the repeated measurements, is a sophisticated method and in those cases it is less urgent to estimate missing data [51], if subjects is missing the remaining available data will be used.

From the start of the cohort there were 309 individuals that chose not to participate. We do have an indication, as we have information from more than half of these individuals; they are not different from the individuals that chose to participate in the study. One other explanation could be a larger number of immigrants are working within HSOs; these individuals could experience language barriers. Further information is that many of the individuals lived in a segregated area where study participation is known to be lower.

There were variables that could have been confounding factors such as age, time of sick leave, pain, and different work tasks. However the research group is homogeneous in regards to sex, all individuals had the same employer, and all were on sick leave, albeit they differ in age, but all age groups are represented. Work ability is considered an age free item [52, 53], the Swedish version of the WAI questionnaire is valuable and suitable to use among different age groups among the working population.

Further result is that our two outcomes of work ability, the full WAI and the work ability score, indicated the same results in the groups with workplace rehabilitation and supportive conditions at work. This result indicates that work ability score can be used as a complement to the full WAI score as an outcome measure among women on long-term sick leave, as we have shown in another study [26].

There might be a challenge to remember and accurately recall workplace conditions for those that have been on sick-leave for a longer time. However, only one of five (19 %) were at full-time degree of sick leave at all the three follow-ups; baseline, 6 and 12 months.

Early assessment of personal resources and hindrances is important in order to increase success in the RTW process [50]. One randomized controlled study showed that participants with upper-extremity musculoskeletal disorder took fewer sick leaves when interventions commenced early [54]. It is crucial to have an effective RTW process for women working within HSO as persistent long-term sick leave both affects personal wellbeing and constitutes a critical social problem [55]. Professionals and employees working with individuals on long-term sick leave need to take actions in the RTW process to determine what is of importance for the individual and to properly time the RTW and involvement of workplace [56].

## Conclusion

The results highlight the importance of integrating workplace rehabilitation with supportive conditions at work in order to increase work ability and improve the RTW process for women on long-term sick leave.
